# Community Attitudes and Practices of Urban Residents Regarding Predation by Pet Cats on Wildlife: An International Comparison

**DOI:** 10.1371/journal.pone.0151962

**Published:** 2016-04-06

**Authors:** Catherine M. Hall, Nigel A. Adams, J. Stuart Bradley, Kate A. Bryant, Alisa A. Davis, Christopher R. Dickman, Tsumugi Fujita, Shinichi Kobayashi, Christopher A. Lepczyk, E. Anne McBride, Kenneth H. Pollock, Irene M. Styles, Yolanda van Heezik, Ferian Wang, Michael C. Calver

**Affiliations:** 1 School of Veterinary and Life Sciences, Murdoch University, Murdoch, Western Australia, Australia; 2 Department of Natural Sciences, Faculty of Social and Health Sciences, Unitec Institute of Technology, Auckland, New Zealand; 3 Department of Natural Resources and Environmental Management, University of Hawaii at Manoa, Honolulu, United States of America; 4 School of Life and Environmental Sciences, University of Sydney, Sydney, New South Wales, Australia; 5 College of Bio-resource Sciences, Nihon University, Fujisawa, Japan; 6 School of Forestry and Wildlife Sciences, Auburn University, Auburn, AL 36849, United States of America; 7 School of Psychology, University of Southampton, Southampton, United Kingdom; 8 Department of Applied Ecology, North Carolina State University, Raleigh, NC, United States of America; 9 Graduate School of Education, University of Western Australia, Perth, Western Australia; 10 Zoology Department, University of Otago, PO Box 54, Dunedin, New Zealand; Curtin University, AUSTRALIA

## Abstract

International differences in practices and attitudes regarding pet cats' interactions with wildlife were assessed by surveying citizens from at least two cities in Australia, New Zealand, the UK, the USA, China and Japan. Predictions tested were: (i) cat owners would agree less than non-cat owners that cats might threaten wildlife, (ii) cat owners value wildlife less than non-cat owners, (iii) cat owners are less accepting of cat legislation/restrictions than non-owners, and (iv) respondents from regions with high endemic biodiversity (Australia, New Zealand, China and the USA state of Hawaii) would be most concerned about pet cats threatening wildlife. Everywhere non-owners were more likely than owners to agree that pet cats killing wildlife were a problem in cities, towns and rural areas. Agreement amongst non-owners was highest in Australia (95%) and New Zealand (78%) and lowest in the UK (38%). Irrespective of ownership, over 85% of respondents from all countries except China (65%) valued wildlife in cities, towns and rural areas. Non-owners advocated cat legislation more strongly than owners except in Japan. Australian non-owners were the most supportive (88%), followed by Chinese non-owners (80%) and Japanese owners (79.5%). The UK was least supportive (non-owners 43%, owners 25%). Many Australian (62%), New Zealand (51%) and Chinese owners (42%) agreed that pet cats killing wildlife in cities, towns and rural areas was a problem, while Hawaiian owners were similar to the mainland USA (20%). Thus high endemic biodiversity might contribute to attitudes in some, but not all, countries. Husbandry practices varied internationally, with predation highest where fewer cats were confined. Although the risk of wildlife population declines caused by pet cats justifies precautionary action, campaigns based on wildlife protection are unlikely to succeed outside Australia or New Zealand. Restrictions on roaming protect wildlife and benefit cat welfare, so welfare is a better rationale.

## Introduction

Cats (*Felis catus*) are widely kept as companion animals [1, 2] and their popularity as pets is increasing in many countries [3, 4]. For example, in Australia, the UK and New Zealand, the proportions of households with a cat are 23% [5], 26% [6] and 35% [7] respectively. Cats have been introduced to most islands and continents across the world, where as pets they are often maintained at high population densities (e.g. > 100/km^2^ [8, 9]).

Pet ownership, including cats, confers numerous benefits to pet-owners but also creates problems for wider society. Benefits include better health and social connection of owners [10–13], as well as opportunities to teach children responsibility, respect and compassion [14–16]. The contribution of pet ownership to national economies through sales of pet food, accessories and veterinary care is also considerable (e.g. [5]). On the other hand, problems arise when cats roam without restriction. These include (i) unwanted hunting of wildlife [7, 17, 18], (ii) transmission of disease to humans, livestock and wildlife [19–21], (iii) potential hybridisation with native wildcats (eg. in Europe [22, 23, 24]), (iv) interbreeding with feral populations, and (v) nuisance to neighbours by fouling yards, harassing caged birds, fighting, spraying and jumping on cars [25, 26]. Roaming cats also risk injury or death [27, 28] and these events are often financially and emotionally costly to owners [29].

Given that pet cats are an important and beneficial part of many people's lives and lifestyles, the most productive approach to ameliorate these problems is to regulate cat husbandry practices to improve cat welfare, reduce nuisance and protect wildlife, while allowing people the pleasure of owning a cat. Tagging (e.g. microchipping) would improve the return of lost and injured animals as well as helping to identify specific nuisances. Desexing (except cats approved for breeding) would reduce the incidence of unwanted kittens, hybridisation with native felids and breeding with feral cats. Likewise, restricting wandering behaviour would decrease predation of wildlife, the spread of disease and traffic accidents involving cats. Understanding the attitudes of the general population towards cat husbandry, as well as the practices of owners, allows governing authorities to create effective regulations sensitive to local situations that are more likely to be accepted, and identifies areas where targeted education may encourage compliance.

Over the last 15 years, several studies have collected data on citizens’ attitudes and practices with regard to cats (both owned and feral) and proposed regulations in several countries, including Australia [30, 31], the USA [32–35], the UK [36] and NZ [37]. While surveys have differed in their questions, timing of administration and sample populations, and were often geographically restricted in each country, the data suggest marked differences between nations in attitudes and practices towards cats. For example, the incidence of confinement of pet cats ranges from 35% [38], quoting data collected in 1997) to 60% [39] in mainland USA, compared to < 10% in Australia [31, 40, 41] or the UK [9]. The prevalence of desexing is consistently > 90% in Australian studies [31, 40, 42, 43] and UK studies [36], compared to c. 80% in the USA [44, 45] or 43% in parts of Italy [46]. Moreover, Australian citizens, including cat owners, also seem more accepting that cats may be a threat to urban wildlife than UK citizens (contrast [30] and [31] with [36]).

Given the variability across nations in how cats are treated and perceived, we sought to test if this variability was an artefact of differences in survey methodology or a true difference, and to greatly extend geographical coverage. We assessed international differences in attitudes and husbandry regarding restrictions and desexing of pet cats, as well as interactions between cats and wildlife, by administering a common survey to cat owners and non-owners in Australia, China, Japan, New Zealand, the UK and the USA. This approach allowed us to compare the attitudes of owners and non-owners in each country to questions such as the desirability of legislation, support for desexing and confinement, and the level of concern over predation by pet cats. We also assessed national variations in response to these questions. While the survey was predominantly exploratory, we also tested explicit predictions that: (i) cat owners would agree less that cats might threaten wildlife than non-cat owners, (ii) cat owners would value wildlife less than non-cat owners, (iii) respondents from Australia, China, New Zealand and the US state of Hawaii (all with high levels of endemic (distinct) wildlife biodiversity) would be more concerned about the potential impacts of pet cats on wildlife than respondents from the UK, the mainland USA and Japan, and (iv) cat owners would be less accepting of cat legislation/restrictions than non-owners. A clearer understanding of citizens' attitudes will be helpful in deciding what, if any, legislative or community education steps might be acceptable in different countries to address perceived problems of predation by pet cats on wildlife.

## Materials and Methods

### Ethics Statement

The Murdoch University Human Ethics Committee (permit 2012/195), the University of Sydney Human Research Ethics Committee (approval no. 15508), University of Hawaii (Manoa) Human Studies Program CHS#20333, University of Southampton Psychology Ethics Committee (Ethics ID: 5775), and University of Otago Human Ethics Committee (Approval D11/297) all approved this study. Written consent was obtained from participants via completion of the first item of the survey form, which also gave documentary evidence of consent. Participants who declined to provide consent did not proceed past the first item.

### Choice of Countries and Cities

The English-speaking nations share common cultural origins despite their current social and political diversity, while Japan is a developed Asian country and China a rapidly developing one. Australia, New Zealand, China and the USA state of Hawaii all have high endemic biodiversity compared to the other countries. We controlled the possibility that attitudes within countries might vary by including at least two cities in each country, where possible across a climatic range (Table 1). In the USA the survey was distributed in two mainland cities (Los Angeles and Chicago) and the Hawaiian Islands, which have significant issues regarding conservation of endemic fauna. In Japan, respondents from Tokyo and Kanagawa were combined into the Japan Capital Area and respondents from the Japanese city of Osaka were combined with small numbers of respondents from other locations to form ‘Japan Other’. The Japanese city Shizuoka was the third city from Japan. Our focus on cities reflects the increasing trend to urbanisation globally.

**Table 1:** List of participating countries and the participating cities from each country, with details of local climate, survey timing and response rates.

Country	Cities—Response rates (no. surveys returned/(no. sent—no. undeliverable)) are in parentheses	Climate	Survey Timing
Australia	Sydney (2.7%), Wollongong (5.3%)	Sydney: Warm temperate, summer highs average 27–30°C and winter highs 17–21°C	Dec 2012 –Mar 2013
		Wollongong: Oceanic, summer highs average 26°C and winter highs 17°C	
New Zealand	Auckland (6.7%), Dunedin (15.9%)	Auckland: Oceanic, summer highs average 24°C and winter highs 14°C	Nov 2012 –Feb 2013
		Dunedin: Oceanic, summer highs average 19°C and winter highs 10°C	
United States of America	Los Angeles (2.9%), Chicago (3.0%), Hawaii (6.8%)	Los Angeles: Mediterranean, summer highs average 29°C and winter highs 20°C	May–July 2013
		Chicago: Humid continental, summer highs average 29^°^C and winter highs 0^°^C	
		Hawaiian islands: Tropical, summer highs average 29–32°C and winter highs 26–28°C	
United Kingdom	Southampton (5.6%), Birmingham (2.6%)	Southampton: Oceanic, summer highs average 22°C and winter highs 8.4°C	Aug–Oct 2012
		Birmingham: Temperate maritime, summer highs average 22°C and winter highs 6.5°C	
Japan	Japan Capital Area, Shizuoka, Japan Other (36.9%)	Tokyo: temperate with four distinct seasons, summer highs average 31°C and winter highs 6°C	July–Nov 2013
		Shizuoka: temperate with four distinct seasons, summer highs average 24°C and winter highs 11°C	
China	Beijing, Harbin (47.1%)	Beijing: Humid continental, summer highs average 31°C and winter highs 2°C	July–Nov 2013
		Heilongjiang: Monsoon influenced humid continental, summer highs average 26°C and winter highs -12°C	

### Administration and Design of Survey

#### Frame, Sampling Design and Contact Method

The survey was administered from spring to autumn in each country when cat activity and prey availability are likely to be high. Temporal effects were controlled by administering the survey in all countries within a 12 month period (Table 1).

For cities in all countries except China, invitations to participate were distributed amongst suburbs with a broad age range of citizens and a high proportion of employed people (i.e. a middle to upper-middle socio-economic demographic more likely to respond to an online survey [47]). These people are also more likely to be politically engaged and hence more vocal in any discussions regarding regulation of the husbandry of pet cats [48, 49]. Within the chosen demographic in each city, 2,000 individuals were selected using simple random sampling without replacement from electoral rolls (New Zealand, UK) or marketing databases (Australia, USA) as the sampling frame. The survey administration method was online for reasons of cost, speed of analysis, alleviating problems with deciphering handwriting, and convenience of reply for the respondents [50]. A personalised invitation letter was sent to all people selected with details of the online survey and an option for requesting a hard copy survey by mail, with a postage-paid reply envelope for its return. A reminder letter was sent two weeks later.

In Japan, 800 invitations to participate in an online survey were distributed at veterinary clinics and local shops (distribution of invitations at shops mitigated the probable bias that clients at veterinary clinics would own a pet of some kind) within suburbs matching the chosen demographic rather than mailed, because the local researchers believed that this was likely to elicit higher responses. Sakurai and Jacobson [51] reported that mailed surveys in Japan rarely exceed response rates of 20–40%, and have been declining steadily since the 1970s. No follow-up was possible in this case. The researchers in China considered it very unlikely that Chinese nationals would respond to an unsolicited online survey from an unknown source: recent Chinese surveys often use interviews [52] or distribute questionnaires to assembled groups [53]. Instead, hired surveyors approached 500 people in Beijing to complete the survey. In Harbin, 500 people known to the researchers by acquaintance, and matching the chosen middle to upper-middle socio-economic demographic, were contacted directly by email and asked to return a completed survey. The decision to use convenience samples rather than probability samples in Japan and China was a trade-off between possible aversion to the probability sample approach in those countries and the lack of consistency in approaches across all countries.

#### Questionnaire Design

The survey was based on that developed by Grayson, Calver and others [30] and adapted by Lilith, Calver and others [31], with the goal of determining public opinion on aspects of cat husbandry, predatory interactions between pet cats and wildlife, and legislative regulation of cat ownership. For our study additional items were added to strengthen assessment of respondents' attitudes to interactions between cats and wildlife, and to restrictions on cat ownership or husbandry. Minor changes to the wording of some items occurred between countries in order to address differences in colloquial terms. There were 77 items overall, 44 assessing opinions and 33 assessing the characteristics of respondents and, for owners, their cat husbandry practices. Items were a mix of direct questions and responses on a five-point Likert scale (Strongly agree, agree, disagree, strongly disagree, I don't know). No item in the online survey insisted on a response, because this might have led to respondents abandoning the survey [54]. However, it did cause variations in response rates for individual items. A copy of the Australian version of the survey is available from the corresponding author on request. Surveys for Japan and China were translated by the authors from those countries.

Eight key items (scored on a five-point Likert scale) were selected for individual analysis to provide insights into the attitudes and beliefs of owners and non-owners in each country on specific issues. These were:

- There is a need for cat legislation
- All cats should be kept in at night time
- Cats should be kept on their owner's property at all times
- It is important to have wildlife in cities, towns and rural areas
- Pet cats killing wildlife in cities, towns and rural areas is a serious problem
- Pet cats on farms are harmful to wildlife
- Pet cats in nature reserves are harmful to wildlife
- Except for a cat owned by a breeder, all cats should be desexed

Further questions relating only to owners were examined to determine differences in husbandry between countries and whether or not the cats had a history of catching wildlife:

- How many cats do you currently own?
- Has this cat been desexed?
- Does this cat live:
  - Solely inside
  - Solely outside
  - Solely inside during the night, but free roaming during the day
  - Inside and outside, but restricted to my property
  - Inside and outside, but free roaming
- Has this cat ever caught anything?

Using the Rasch measurement model [55, 56], three scales were constructed based on responses to the items on attitudes and practices: 1) Restrictions, dealing with regulations on cat ownership; 2) Wildlife, considering interactions between pet cats and wildlife; and 3) Desexing, covering issues related to desexing pet cats (see below for details). Respondents' scores on these scales were used as dependent variables indicating their attitudes.

The survey program iSurvey, from the University of Southampton, was used by respondents from Australia, New Zealand, the USA and the UK, with each country having its own customised survey and login. The Japanese survey used Survey Monkey (https://www.surveymonkey.com). Results from China were compiled manually. Any paper surveys received were entered manually.

### Data Analysis

#### Response Rates and Representativeness of the Survey

Response rates, defined as the number of surveys completed either online or by paper divided by the number of invitations sent minus the number returned as undeliverable [57], were calculated for each city where invitations were mailed. In Japan, response rates were calculated as the number of surveys completed divided by the number of leaflets distributed, while in China they were calculated as the number of people responding divided by the number approached. Responses collected online may not be representative [50], so we tested the representativeness of the samples by: (i) comparing the proportions of cat owners in the responses for each country with recent independent assessments of the proportion of cat ownership in those countries, (ii) checking for non-response bias by comparing the responses of people responding promptly to those responding tardily to the survey, and (iii) comparing mailed and online responses. These measures apply only to our target middle to upper-middle socio-economic demographic and cannot be extrapolated beyond it.

We compared the proportions of cat owners and non-owners in the study to estimates of cat ownership in each country from data published within the last decade (Australia 23% [5]; NZ 35% [7]; UK 26% [6]; USA 30% [58]; Japan 10% [59]; China 15% [60]. We used chi-squared goodness of fit tests with continuity correction to determine whether the relative proportions of cat owners to non-owners who responded were equivalent to the relative proportions in the general population for each country.

Armstrong and Overton [61] argued that people who respond less readily to surveys, as indicated by a tardy response or a response only after prompting, are more likely to have similar attitudes to non-respondents. Therefore, if there are differences in characteristics or answers between prompt and tardy respondents non-response bias is likely, requiring a correction. We divided the respondents into early (responding within two weeks of the return of the first response) and late (responding after two weeks from the first response). This was undertaken on data for Australia, New Zealand, the UK and the USA as information on when the survey was completed was available. Those who completed a paper survey were excluded. Information on the timing of responses was unavailable from the Japanese data and not applicable to the Chinese data.

For each country separately, combining the results for cities within countries, we used a two-way chi-square contingency table with Yates' correction to determine if there was any difference between early and late respondents for i) the proportions of owners to non-owners and ii) the proportions of men to women. Secondly, we tested for differences in the average age between early and late respondents using either a two-tailed *t*-test after confirming homoscedasticity, or a two-tailed *t*-test for heteroscedastic samples. Thirdly, we used log-linear three-way contingency tables to test for associations between agreement (the proportion of respondents agreeing or strongly agreeing to an item), ownership (owners and non-owners) and promptness (whether the respondents answered early or late to the eight specific items above) in each country. Fourthly, for each country we correlated respondents' scores on the Restriction, Wildlife and Desexing scales with the length of time they took to respond (measured in days from the date of initial mailing of the invitation to participate). Correlations significant at *p* < 0.05 were interpreted as evidence of non-response bias.

We also tested for differences between online and paper surveys. There were too few paper surveys from Australia and the UK to analyse, so we analysed only respondents from New Zealand and the USA. Countries were analysed separately and cities within countries were combined.

We used two-way chi-square contingency tables with Yates' correction to evaluate associations between whether people responded by mail or online and the relative proportion of owners and non-owners, men and women, and employment status (working, retired or unemployed). We tested for differences in age between mail and online respondents using either a two-tailed *t*-test after confirming homoscedasticity or a two-tailed *t*-test for heteroscedastic samples. We also used log-linear three-way contingency tables to test for associations between agreement (strongly agree and agree combined)/disagreement (strongly disagree and disagree combined) to the eight specific items above, owners/non-owners, and online/paper survey.

#### Analysis of Specific Items for All Respondents

We divided all responses simply into agree or disagree to avoid problems caused by limited responses in some of the finer categories, as well as avoiding problems of cultural differences in preferences for selecting middle or extreme values [62]. Respondents who answered “I don’t know” to a particular item were excluded from analysis for that item only.

For each item, we used chi-squared homogeneity tests [63] to determine whether the proportion of agreement for owners and non-owners between cities in the same country was the same and therefore whether the data for the cities within each country could be pooled. Those respondents who did not indicate what city they were from were excluded from this analysis. We then used a Generalized Linear Model (GLM) in Statistica 12 [64] to assess relationships between the predictor variables (Country, Cat ownership (i.e. cat owners and non-owners) and the Country x Cat ownership interaction) and the dependent variable of agreement with the statement. As there were only two possible answers to each question (agree/disagree), we evaluated the binomial distribution with a logit link function. For countries where the cities were homogeneous according to the previous test, the data were pooled. If not, the cities of that country were analysed separately for that item only. If the cities were considered homogeneous, data from respondents who did not indicate which city they were from were included in the totals for their country. If cities were not homogenous for an item, these respondents were excluded for that item.

#### Analysis of Specific Items for Cat Owners

For each of the items specific to cat owners we used chi-square contingency tables to evaluate if (i) there were any differences between cities in the same country and therefore whether data could be pooled, and (ii) whether there were any differences between countries. If cities within countries were not significantly different at the 0.05 level, respondents who answered these items but did not disclose what city they were from were included in the totals for that country. Otherwise, they were excluded. For the question ‘how many cats do you currently own?’ responses were divided into 1 cat, 2 cats and > 2 cats because few owners owned more than two cats.

The survey asked owners to provide information on up to four of their cats if applicable. Information for all of the cats mentioned was used in the analyses. For example, for the question ‘has this cat been desexed?’, if an owner provided information on three cats, all three cats were recorded and contributed to the total sample size.

#### Construction of Rasch Scales

The Rasch measurement model was used to establish the psychometric properties of three scales (Restriction, Wildlife and Desexing) using RUMM2030 [65]. This examines the fit of a set of data to a linearised uni-dimensional model, which, if the data fit the model, places survey questions and respondents’ attitudes relative to one another on a single equal-interval continuum. This produces locations (scores) for each survey item and every respondent. These locations are directly comparable with each other and, since they are linearised, are more appropriate for use in common statistical tests than raw scores. Respondents scoring more highly on the Restriction scale were more supportive of cat legislation, including items such as limiting the number of cats that can be owned per household or opportunities for cats to roam. Those scoring more highly on the Wildlife scale were more likely to be concerned about negative impacts of roaming cats on wildlife, while respondents scoring more highly on the Desexing scale were more knowledgeable about desexing and cat behaviour, more supportive of desexing their own pet cats, and more supportive of requiring others to do likewise.

#### Analysis of the Rasch Person Locations on the Three Scales

Each of the three scales was analysed separately as a dependent variable in a nested GLM using Statistica 12 [64]. Country, City (nested within country), Cat ownership status and the Country x Cat ownership interaction were used as predictor variables to test relationships with the dependent variables. We did not extend the analysis to consider, for example, differences in responses between men and women or between people of different ages because inclusion of large numbers of variables in relation to sample size risked overfitting in statistical models. Significance levels for the tests were set at *p* < 0.01 to compensate for heteroscedasticity that could not be corrected by logarithmic transformation [66]. Respondents who did not indicate their city were excluded from these analyses. However, if city was not a significant predictor alone or in interaction, we then repeated the analyses excluding city as a predictor and included respondents who did not give a city.

## Results

### Representativeness of the Survey and Non-Response Bias

#### Characteristics of Survey Respondents

In the presentation of results that follows and in the discussion we refer simply to categories of respondent by country and by cat ownership status, without reiterating that our respondents belong to a specific middle class demographic. They cannot be considered representative of other demographics in the populations of these countries.

There were 1720 respondents across the six countries. Most responses were from China (471–47.1% response rate) followed by New Zealand (347–11.5% response rate), Japan (295–36.9% response rate), the USA (282–5.0% response rate), Australia (169–4.3% response rate) and the UK (156–3.9% response rate). More women responded to the survey than men in all countries except the USA. On average, the respondents from Australia, the UK, New Zealand and the USA were in their 50s. Respondents from Japan and China were much younger with average ages of 31 and 36 respectively (Table 2).

**Table 2:** Characteristics of respondents in each country.

City and Country	n	Male	Female	Owner	Non-owner	Mean age^1^	Early^2^	Late^3^	Online^4^	Mail^5^
Sydney	53	25	28	11	42	56±13	31	20	51	2
Wollongong	108	54	54	22	86	60±14	60	45	105	3
Unspecified	8									
Australia Total	169	79	82	34	132	59±14	91	65	156	5
Auckland	99	42	57	53	46	48±16	56	36	92	7
Dunedin	225	84	141	114	111	53±16	126	61	187	38
Unspecified	23									
New Zealand Total	347	126	203	175	164	52±16	182	97	279	45
Chicago	62	42	20	18	44	54±13	27	33	60	2
Los Angeles	61	33	28	26	35	54±14	23	35	58	3
Hawaii	140	91	48	42	98	56±14	65	56	121	18
Unspecified	19									
USA Total	282	167	101	91	182	55±14	115	24	239	23
Southampton	107	52	54	42	65	50±18	65	47	105	7
Birmingham	49	17	32	15	34	52±18	27	22	51	0
Unspecified	0									
UK Total	156	69	86	57	99	51±18	207	193	156	7
Japan Capital Area	87	32	55	17	70	28±8	N/A	N/A	N/A	N/A
Shizuoka	101	25	75	36	65	38±14	N/A	N/A	N/A	N/A
Japan Other	65	15	50	16	48	25±13	N/A	N/A	N/A	N/A
Unspecified	42									
Japan Total	295	72	181	82	190	31±13	N/A	N/A	N/A	N/A
Beijing	143	147	148	53	220	37±16	N/A	N/A	N/A	N/A
Heilongjiang	305	49	90	6	115	34±15	N/A	N/A	N/A	N/A
Unspecified	23									
China Total	471	203	245	61	350	36±15	N/A	N/A	N/A	N/A

^**1**^Mean ± standard error.

^2^Responded within two weeks of invitation.

^3^Responded more than two weeks after invitation.

^4^Responded online.

^5^Requested a hard copy survey and responded by mail.

#### Proportions of Cat Owners

In Australia, the USA and China cat owners were represented in the sample in the same proportions as expected based on ownership for the population (*p* ≥ 0.22 in all cases). In New Zealand, the UK and Japan, cat owners were over-represented in the sample (χ^2^_1_ = 30.11, *p* <0.0001, χ^2^_1_ = 10.04, *p* = 0.002 and χ^2^_1_ = 119.57, *p* < 0.0001, respectively; Table 2).

#### Non-Response Bias

The proportions of owners and non-owners, men and women, and age categories did not vary depending on whether people responded early or late from each country (*p* > 0.10 in all cases). Similarly, agreement/disagreement with seven of the eight specific items was not associated with whether people responded early or late. The exception was 'All cats should be kept in at night time', where late cat owners in Australia were more likely to agree (G^2^_2_ = 6.32, *p* = 0.042), while late cat owners in the USA were less likely to agree (G^2^_2_ = 6.2, *p* = 0.045). These trends were borne in mind when interpreting the analysis of this item. Non-response bias was not detected in the other questions. No significant correlations were found between respondents' scores on the Restriction, Wildlife and Desexing scales and the promptness with which they responded to the survey (*r* ≤ 0.215 in all cases), so there was no evidence of non-response bias in these scales.

Given the almost total absence of evidence for non-response bias for Australia, New Zealand, the UK and the USA, we assumed no non-response bias for Japan (where individual survey timing information was unavailable) and in China, which had the highest overall response rate. Undetected non-response bias may exist, but with no evidence of the direction in which this might be operating no correction was possible.

#### Mail Survey Respondents

Similar proportions of owners and non-owners (NZ: χ^2^_1_ = 0.01, *p* = 0.92; USA: χ^2^_1_ = 0, *p* = 1) and men and women (NZ: χ^2^_1_ = 0.11; *p* = 0.74; USA: χ^2^_1_ = 0.88, *p* = 0.35) responded online or by mail. However, there were significantly more retired people in both New Zealand and the USA who responded by mail (*p* ≤ 0.0001 for both countries), with mail respondents significantly older by about 20 years in New Zealand and 10 years in the USA than online respondents (*p* < 0.0001 for both countries). Mail survey respondents from the USA were more likely to agree 'That there is a need for cat legislation' (G^2^_2_ = 6.8, *p* = 0.03) and disagree with 'It is important to have wildlife in cities, towns and rural areas' (G^2^_2_ = 9.24, *p* = 0.01). New Zealand mail survey respondents were more likely to agree that 'Except for a cat owned by a breeder, all cats should be desexed' (G^2^_2_ = 8.48, *p* = 0.01). There were no significant differences in responses for the other specific items. Online and mailed responses were pooled for analysis of specific items and development of Rasch scales.

### Responses to Specific Items for All Respondents

For most of the specific items, cities within countries were deemed homogenous with only three exceptions. For 'There is a need for cat legislation' and 'Except for a cat owned by a breeder, all cats should be desexed', Hawaii was significantly different from Los Angeles and Chicago. In these instances, Hawaii was treated as a separate country but Los Angeles and Chicago were pooled to form mainland USA (after passing the homogeneity test). The cities within Japan were all significantly different for 'It is important to have wildlife in cities, towns and rural areas' and were treated separately for this item.

#### There is a Need for Cat Legislation

**Fig 1:**
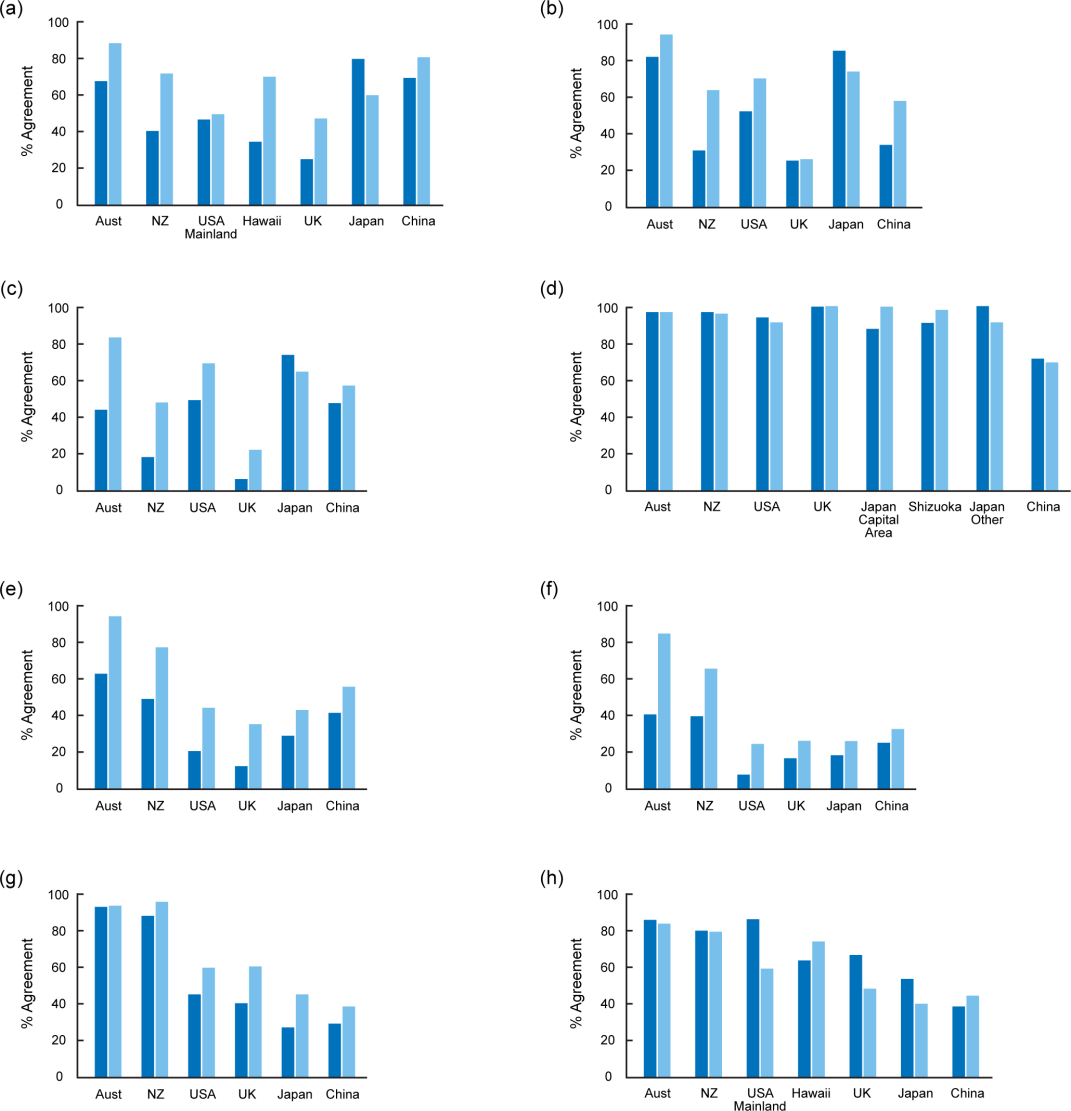
Percentage agreement of cat owners (dark blue) and non-owners (light blue) in each country to eight survey items: (a) There is a need for cat legislation (b) All cats should be kept in at night time (c) Cats should be kept on their owner's property at all times (d) It is important to have wildlife in cities, towns and rural areas (e) Pet cats killing wildlife in cities, towns and rural areas is a serious problem (f) Pet cats on farms are harmful to wildlife (g) Pet cats in nature reserves are harmful to wildlife (h) Except for a cat owned by a breeder, all cats should be desexed.

There were significant effects for country, ownership and the country x ownership interaction (Table 3 and S1 Table). Non-owners were more supportive of the need for cat legislation than owners everywhere except in Japan (Fig 1A). Australian non-owners were the most supportive (88%) followed by Chinese non-owners (80%) and Japanese owners (79.5%). The UK respondents showed least agreement, especially cat owners (25%). The difference between cat owners and non-owners was most marked in New Zealand and Hawaii; conversely there was almost no difference in the results between owners and non-owners on the mainland USA (Fig 1A).

**Table 3:** Results of analysis of specific survey questions. Cities within countries are combined, unless responses were shown to differ between cities.

Question	Countries	GLM result				Interpretation
			Wald Chi-square	d.f.	Sig.	
There is a need for cat legislation	Australia, New Zealand, UK, USA Mainland, Hawaii, Japan, China	Intercept	36.696	1	<0.001	Non-owners were more supportive of the need for cat legislation than owners everywhere except in Japan.
		Country	81.173	6	<0.001	
		Ownership	23.061	1	<0.001	
		Country*Ownership	35.790	6	<0.001	
All cats should be kept in at night time	Australia, New Zealand, UK, USA, Japan, China	Intercept	37.651	1	<0.001	Owners were less supportive than non-owners, except in Japan where owners were more supportive and in the UK, where owners and non-owners had similarly low agreement.
		Country	142.813	5	<0.001	
		Ownership	16.112	1	<0.001	
		Country*Ownership	25.651	5	<0.001	
Cats should be kept on their owner's property at all times	Australia, New Zealand, UK, USA, Japan, China	(Intercept)	2.07	1	0.150	Owners were generally less supportive than non-owners except in Japan, where this was reversed.
		Country	130.148	5	<0.001	
		Ownership	35.159	1	<0.001	
		Country*Ownership	31.005	5	<0.001	
It is important to have wildlife in cities, towns and rural areas	Australia, New Zealand, UK, USA, Japan Capital Area, Shizuoka, Japan Other, China	(Intercept)	0.000	1	0.997	Support for retaining wildlife in settled areas attracted strong agreement irrespective of cat ownership.
		Country	75.670	7	<0.001	
		Ownership	0.000	1	0.999	
		Country*Ownership	2.945	7	0.890	
Pet cats killing wildlife in cities, towns and rural areas is a serious problem	Australia, New Zealand, UK, USA, Japan, China	(Intercept)	0.946	1	0.331	Non-owners were more supportive than owners in all countries, although in Australia 62% of owners agreed.
		Country	123.967	5	<0.001	
		Ownership	55.927	1	<0.001	
		Country*Ownership	10.002	5	0.075	
Pet cats on farms are harmful to wildlife	Australia, New Zealand, UK, USA, Japan, China	(Intercept)	80.946	1	<0.001	In all countries, owners were less likely to agree than non-owners although all respondents from Australia and New Zealand, regardless of ownership, were more likely to agree than respondents elsewhere.
		Country	113.130	5	<0.001	
		Ownership	33.847	1	<0.001	
		Country*Ownership	11.461	5	0.043	
Pet cats in nature reserves are harmful to wildlife	Australia, New Zealand, UK, USA, Japan, China	(Intercept)	52.070	1	<0.001	Owners were less likely to agree with this item than non-owners. Support was very high in Australia and New Zealand, weaker in the USA and the UK, and lowest in Japan and China.
		Country	187.618	5	<0.001	
		Ownership	10.929	1	<0.001	
		Country*Ownership	2.409	5	0.790	
Except for a cat owned by a breeder, all cats should be desexed	Australia, New Zealand, UK, USA Mainland, Hawaii, Japan, China	(Intercept)	80.082	1	<0.001	In each country agreement was generally higher for this item from cat owners, excepting Hawaii and China, where non-owners were more supportive.
		Country	113.569	6	<0.001	
		Ownership	4.486	1	0.034	
		Country*Ownership	14.884	6	0.021	

* Indicates the Country by Ownership interaction.

#### All Cats Should be Kept in at Night Time

There were again significant effects for country, ownership and the country x ownership interaction (Table 3 and S1 Table). Generally, owners were less supportive than non-owners, except in Japan where owners were more supportive and in the UK, where owners and non-owners had similarly low agreement (Fig 1B). Agreement was highest in Australia, followed closely by Japan with over 80% agreement from all Australian respondents and Japanese owners. Support was lowest in the UK, with respondents showing less than 30% agreement irrespective of cat ownership.

#### Cats Should be Kept On Their Owner’s Property At All Times

There were significant effects for country, ownership and the country x ownership interaction (Table 3 and S1 Table). Owners were generally less supportive than non-owners except in Japan, where this was reversed (Fig 1C). Australian non-owners were the only group that showed above 80% agreement, while lowest agreement was for New Zealand owners (18.6%), and both owners (6.9%) and non-owners (22.7%) in the UK.

Although both this item and the previous one consider restricting cat wandering behaviour, confining cats to their owners' properties at all times was less popular amongst the majority of respondents, with the exception of owners and non-owners from the USA and Chinese non-owners where responses stayed approximately the same. The differences between cat owners and non-owners were also much stronger for this item except in the USA and Japan, where the differences remained about the same (Fig 1C).

#### It Is Important to Have Wildlife in Cities, Towns and Rural Areas

There was a significant effect of country but no effect of ownership or the country x ownership interaction (Table 3 and S1 Table). Support for the retention of wildlife in settled areas was very high and, irrespective of cat ownership, attracted over 85% agreement in all countries except China, where only approximately 65% of respondents agreed (Fig 1D).

#### Pet Cats Killing Wildlife in Cities, Towns and Rural Areas is a Serious Problem

There were significant effects for country and ownership, but no significant effect of country x ownership interaction (Table 3 and S1 Table). Non-owners were more supportive than owners in all countries, although in Australia 62% of owners agreed (Fig 1E). Overall, support for this item was highest in Australia followed by New Zealand and least in the UK, where only 12% of owners and 38% of non-owners agreed.

#### Pet Cats On Farms are Harmful to Wildlife

There were significant effects for country, ownership and country x ownership interaction (Table 3 and S1 Table). In all countries, owners were less likely to agree than non-owners, especially in Australia and New Zealand (Fig 1F). However, all respondents from Australia and New Zealand, regardless of ownership, were more likely to agree with this item than respondents from any other country. Support was lowest from cat owners in the USA (8%). With the exception of owners and non-owners from the UK, support for this item was consistently lower than for 'Pet cats killing wildlife in cities, towns and rural areas is a serious problem' and 'Pet cats in nature reserves are harmful to wildlife.'

#### Pet Cats in Nature Reserves are Harmful to Wildlife

There were significant effects for country and ownership, but not for the country x ownership interaction (Table 3 and S1 Table). Owners were less likely to agree with this item than non-owners (Fig 1G). Support was very high in Australia and New Zealand, with more than 88% of owners and non-owners in each country agreeing that pet cats in nature reserves are harmful to wildlife. The USA and the UK formed a second group with support c. 40% for this item for owners and 60% for non-owners, with Japan and China forming a third group with support c. 30% for owners and 40% for non-owners. For owners and non-owners from Australia, New Zealand, the UK and the USA, support for this item was consistently higher than for 'Pet cats killing wildlife in cities, towns and rural areas is a serious problem' and 'Pet cats on farms are harmful to wildlife.'

#### Except for a Cat Owned by a Breeder, All Cats Should Be Desexed

There were significant effects of country, ownership and country x ownership interaction (Table 3 and S1 Table). In each country agreement was generally higher for this item from cat owners, with the exception of Hawaii and China, where non-owners were more supportive (Fig 1H). Levels of support were highest among cat owners from Australia, New Zealand and the mainland USA, and lower for UK non-owners, Japan and China.

### Responses to Specific Questions for Cat Owners

#### How Many Cats do You Currently Own?

**Fig 2:**
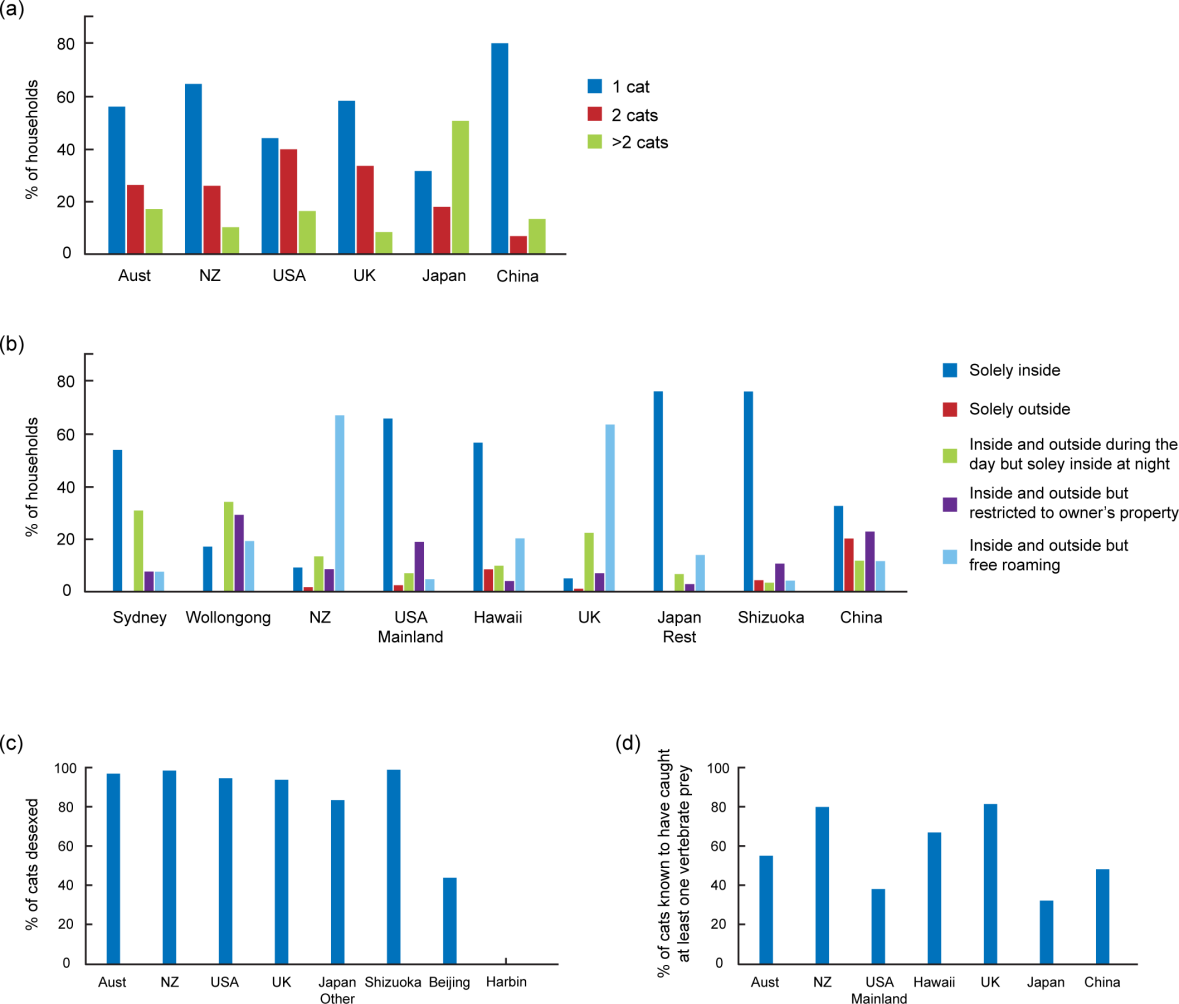
Cat husbandry practices in different countries. (a) Percentage of households that own one, two or more than two cats (b) Percentage of cats kept in different conditions of confinement (c) Percentage of desexed cats. (d) Percentage of cats that have ever caught vertebrate prey.

The number of cats owned by households varied significantly between countries ($\chi_{10}^{2}$ = 92.99, *p* < 0.0001). With the exception of Japan, the largest ownership category was single-cat households (Fig 2A). In China, the proportion of single-cat households was especially high (80%) compared to other countries, with New Zealand next (64%). In the USA, the number of households with only one cat (44%) was only slightly higher than the number of households with two cats (40%). In Australia, New Zealand and the UK there was a drop of at least 25% between one and two cat households. China had a 73% drop between one- and two-cat households. China and Japan had more ‘more-than-two-cats’ households than two-cat households (Fig 2A). Households in the UK were the least likely to have more than two cats (8%). Japan was unusual in that most households (51%) had more than two cats followed by single-cat households (32%) and then households with only two cats (17%) (Fig 2A). Some cat owners in Japan owned very high numbers of cats. Ten households (13%) reported owning 10 or more cats with the highest number being 99 and the next highest 27. Although the 99 could be an error, it might indicate a person claiming ownership of a cat colony.

#### Does this Cat Live: …

This question targeted whether cats were kept either solely inside, solely outside, inside at night but free roaming during the day, inside and outside but restricted to the owner’s property, or inside and outside but free roaming. There was a significant association between confinement and countries ($\chi_{32}^{2}$ = 453.6, *p* < 0.0001). This item was also highly variable within countries, with Australia divided into Sydney and Wollongong, the USA into mainland USA and Hawaii, and Japan divided into Shizuoka and Japan Rest. Cats in Sydney (53%), the mainland USA (66%) and both locations in Japan (75%) were most likely to be kept solely inside (Fig 2B).

Although Sydney and Wollongong were significantly different from each other, cat owners in Wollongong still favoured restricting their cats’ wandering behaviour either by keeping them in at night (34%), or by restricting them to their property (29%). However, owners in Wollongong were more likely to let their cats be inside and outside but free roaming (20%) than owners in Sydney (8%). Cat owners in New Zealand and the UK reported similar patterns: most cats were "free roaming inside and outside" (67% and 64% respectively), followed by cats kept in at night (14% and 23% respectively). On the mainland USA, cat owners favoured restrictions by keeping their cats solely inside (66%), inside and outside but restricted to their property (19%) or inside at night but free roaming during the day (8%). However, in Hawaii, although cats were predominantly kept solely inside (56%), 20% "were free roaming inside and outside". In Japan most cats were kept solely inside (75%), but the second option preferred by Japan Rest was for cats to be inside and outside but free roaming (14%), compared to inside and outside but restricted to their property in Shizuoka (11%). China showed the least variance. Although 32% of cat owners preferred to keep their cats solely inside, even their lowest two preferences of "inside at night but free roaming during the day" (12%) and "free roaming inside and outside" (12%) were more popular than the second preference in Shizuoka, which was "inside and outside but restricted to owner's property" (Fig 2B).

#### Has this Cat Been Desexed?

There was a significant difference between countries in the proportion of cats desexed ($\chi_{7}^{2}$ = 284.4, *p* < 0.0001), and high levels of variability between cities in China and Japan. Shizuoka was separated from the other Japanese localities, which were all combined into Japan Rest. Beijing and Harbin were considered separately. In general, desexing rates were very high (over 94% in Australia, New Zealand, the USA, the UK and Shizuoka (Fig 2C)). Japan Rest had lower desexing rates than Shizuoka (83% and 99% respectively). China had much lower desexing rates than the other countries (43% in Beijing and 0% in Harbin (Fig 2C)).

#### Has this Cat Ever Caught any Vertebrate Prey?

There was a significant difference between countries in the proportion of cats that had been known to catch prey at least once in their lives ($\chi_{6}^{2}$ = 124.1, *p* < 0.0001). The highest proportions were in the UK (82%) and New Zealand (79%), followed by Hawaii (67%; Fig 2D). Respondents from Japan (32%) and the USA Mainland (38%) reported the lowest proportion of cats that were known to catch prey.

### Analysis of the Rasch Person Locations for Three Scales

Overall conclusions about the scales’ internal consistency and reliability are provided. All but two items (R14 and R16) in the Restrictions scale showed good fit to the model and these were deleted from the final scale as they are measuring a different variable. The Person Separation Index (an index of reliability) was high at 0.856, indicating that this scale provides valid and reliable person measures. To obtain good fit to the Rasch model, one item (W11) was deleted from the Wildlife scale, and two items (S5 and S9) from the Desexing scale. Both scales had lower reliability than the Restriction scale (0.589 and 0.605, respectively). They would benefit from a greater range of items to improve their reliability: at present the items are too homogeneous, relative to the respondents. Analysis of a combination of all three scales (with items S5, S9, R14 and R16 deleted) showed they may, for particular research contexts, be considered as a single scale representing attitudes to cat care and control. The Person Separation for the combined scale was high at 0.847. Using the person locations from each of the three scales separately, traditional statistical techniques were carried out as follows.

With a significance level of 0.01, cities within countries gave consistent results for all scales (Restriction: *F*_(8,1525)_ = 1.94, *p* = 0.050), Desexing: (*F*_(8,1476)_ = 1.32, *p* = 0.226), Wildlife: (*F*_(8,1485)_ = 2.24, *p* = 0.022). Therefore analyses were repeated without cities nested within country as a predictor and respondents who did not indicate a city were included.

**Fig 3:**
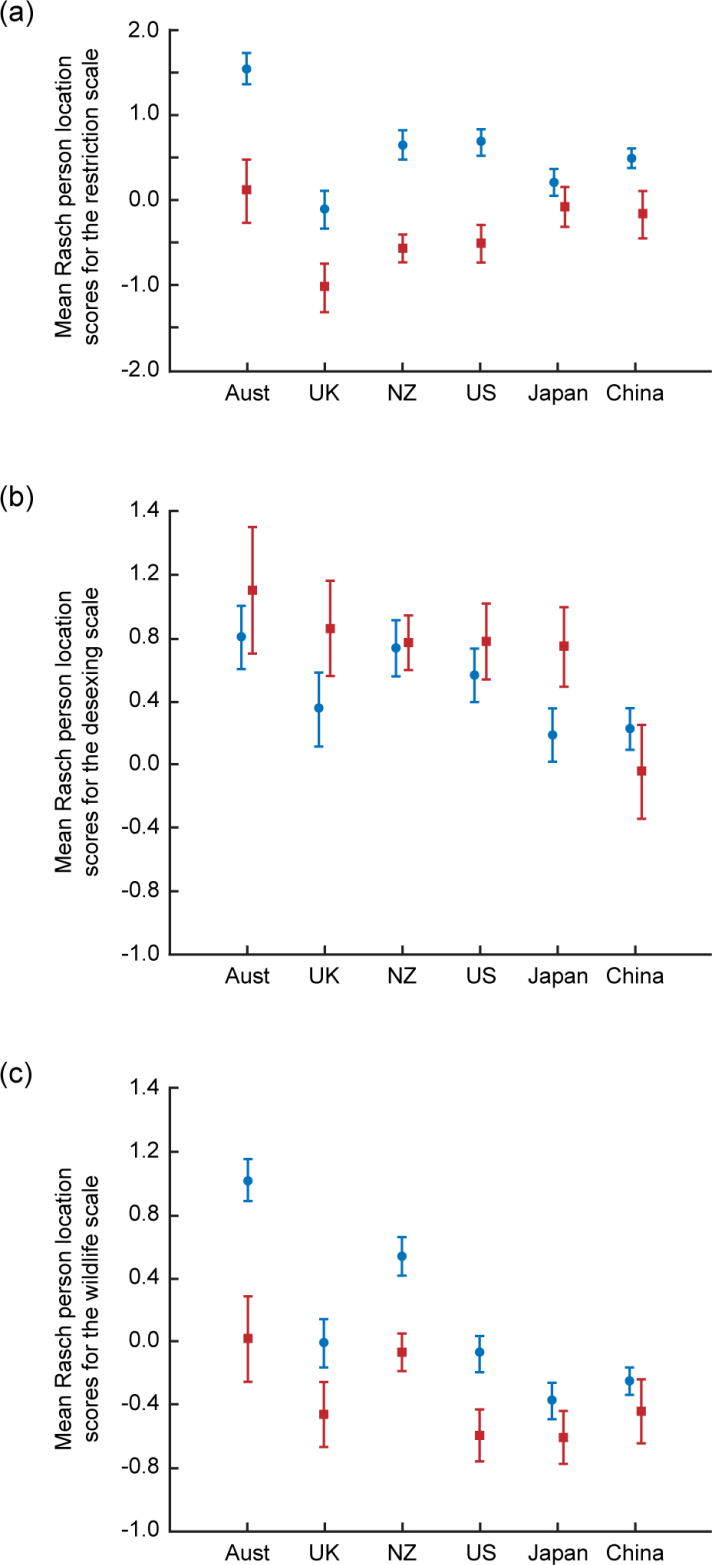
Mean Rasch person location scores, ± 95% confidence limits, for owners (red) and non-owners (blue) on (a) The restriction scale (b) The desexing scale (c) The wildlife scale.

On the Restriction scale, there were significant effects for country (*F*_(5,1599)_ = 20.43, *p* < 0.001), ownership (*F*_(1,1599)_ = 208.53, *p* < 0.001) and the country x ownership interaction (*F*_(5,1599)_ = 7.53, *p* < 0.001). In each country non-owners were more supportive of restrictions than owners. This was especially so in Australia, but much less so in Japan. Australian non-owners were more supportive of restrictions on cats than non-owners from other countries, and the same was true for Australian owners compared to owners elsewhere. Support for restrictions was lowest in the UK. The significant country x ownership interaction was driven strongly by the contrast between Japan, where there was only a very small difference in the opinions of owners and non-owners, and Australia, where there was a large difference between owners and non-owners (Fig 3A and S2 Table).

On the Desexing scale, there were significant effects for country (*F*_(5,1547_ = 11.42, *p* < 0.001), ownership (*F*_(1,1547)_ = 9.97, *p* = 0.002) and the country x ownership interaction (*F*_(5,1547)_ = 4.93, *p* = 0.003). Owners were more supportive of desexing than non-owners except in China, where non-owners tended to be more supportive. Support for desexing was highest in Australia and lowest in China (Fig 3B and S2 Table).

On the Wildlife scale there were significant effects of country (*F*_(5,1555)_ = 45.13, *p* < 0.001), ownership (*F*_(5,1555)_ = 109.26, *p* < 0.001) and the country x ownership interaction (*F*_(5,1555)_ = 5.25, *p* < 0.001). In each country non-owners showed higher scores than owners. This difference was especially marked in Australia and New Zealand, but much less in China and Japan. Internationally, Australian and New Zealand non-owners had the highest scores compared to other non-owners, and the same was true for owners (Fig 3C and S2 Table).

## Discussion

### Tests of Specific Predictions

Our predictions that cat owners would be less accepting of statements implying that cats threaten wildlife than non-owners and be less accepting of cat regulation were largely fulfilled. In all countries non-owners were more likely than owners to believe that pet cats killing wildlife were a problem in a range of locales, while legislation was supported most strongly by non-owners everywhere except in Japan. We have no specific evidence of why owners were less likely to believe that pet cats killing wildilfe was a problem. Where the predominant practice of owners was to confine their pets at all times, this belief likely rests on the sound premise that confined cats cannot hunt wildlife, although wildlife protection need not be the motivation for confinement. In other cases, owners presumably believed that predation by pet cats was an insignificant factor in determining the distribution and abundance of prey species. Alternatively, they chose to value the convenience of their pets over wildlife. However, the prediction that owners valued wildlife less than non-owners was not supported. Significant differences between countries were identified, offering partial support for the prediction that respondents from Australia, New Zealand, China and the US state of Hawaii (all with high endemic wildlife biodiversity) would be more concerned about impacts of pet cats on wildlife than respondents from elsewhere. Even large proportions of Australian (62%), New Zealand (51%) and Chinese owners (42%) agreed that pet cats killing wildlife in cities, towns and rural areas was a problem (although Hawaii matched the mainland USA). Hawaiian non-owners, though, were more supportive of cat legislation and desexing pet cats than non-owners on the mainland USA. Overall, the pattern of responses seems to be determined by a complex of historical and cultural conditions.

### International Differences in Attitudes to Cats and Wildlife

Marked national differences occurred in responses to individual questions and in the analyses of the Rasch scales. These have implications for any attempts to regulate cat ownership in each country. We discuss these in the context of research in different countries that has attempted to quantify any impacts of pet cats on wildlife. We did not extend the analysis to consider, for example, differences in responses between men and women or between people of different ages because inclusion of large numbers of variables in relation to sample size risked overfitting in statistical models. However, these variables may also have an influence.

#### Australia

The popularity of cats as pets in Australia is declining, with the pet cat population estimated at 2.93 million in 1994 and 2.35 million in 2009. The percentage of households owning a cat declined from 24.6% to 22.8% over the same period [5]. The second highest reason for Australians not owning a cat (after dislike of cats) is concern about wildlife [67, 68]. Thus it was unsurprising that Australian owners and non-owners scored highly on the wildlife Rasch scale and were likely to believe that pet cats are harmful to wildlife in cities, towns and rural areas and nature reserves. Most Australian non-owners (85%) were also likely to believe that pet cats are harmful to wildlife on farms, but not owners (41%).

Australians have a special preference for their native fauna compared to citizens from the UK, the USA, India and South Africa [69]. Feral predators such as cats and foxes (*Vulpes vulpes*) are accepted as significant threats to Australian fauna [70, 71], with feral cats now assessed as endangering more threatened and near threatened Australian mammalian taxa than any other factor [72]. Concern about predation by feral cats on wildlife is manifested in significant paintings by contemporary artists and in museum displays, with some extending to predation by pet cats. Such sustained messages in varied media are reflected in high concern by both owners and non-owners that predation by pet cats on wildlife in cities, towns and rural areas endangers wildlife. Such concerns are at least 25 years old [73, 74].

Research on the effects of pet cats on wildlife populations in Australia has resulted in more ambiguity than popular opinion would suggest. Urban habitats provide important refuges for threatened species that are vulnerable to cat predation, including a legless skink (*Delma impar*) in suburban Canberra [75] and the eastern barred bandicoot (*Perameles gunnii*) in Hamilton, Victoria [17]. However, in the case of the eastern barred bandicoot traffic was even more of a threat than predation by cats [17]. Barratt [76] and Grayson, Calver and others [77] concluded that pet cats kill mainly common vertebrates that persist in cities despite predation, although they acknowledged that problems may be more severe near remnant vegetation or on urban fringes. Thus, while pet cats may depress some prey populations, they are also a convenient scapegoat for more intractable causes of wildlife decline [67, 78, 79].

#### New Zealand

Cats are popular household pets in New Zealand, with 35% of households owning at least one [80]. These cats co-exist with a predominantly endemic native fauna, although in urban areas nearly half of bird species and most individuals are exotic [81]. With the exception of bats, there are no native mammals. New Zealand respondents were concerned that their fondness for cats could impact their native wildlife, leading to them having the second highest score on the wildlife scale and high agreement that pet cats are a serious problem for wildlife in cities, towns and rural areas, and in nature reserves. Non-owners were also likely to believe that pet cats are a serious problem on farms. This is consistent with popular cultural messages related to responsible pet ownership such as Crew [82], a children's story recounting the fate of the Stephen's Island wren (*Xenicus lyalli*) at the claws of the lighthouse keeper's cat.

Whether cat husbandry should be regulated to protect wildlife proved more contentious. Although support for legislation amongst non-owners was high (70%), support from cat owners was substantially lower (40%) and New Zealand owners scored the second lowest on the restriction scale after UK owners.

Despite the ambivalence of owners towards restrictions, there is evidence that predation by pet cats in New Zealand is likely to be additive (increasing the overall mortality) rather than compensatory (removing individuals that would otherwise die from other causes) for at least some species of New Zealand birds, with urban populations likely being sinks replenished from source habitats with less predation [7]. A New Zealand study also provides the most comprehensive record of predation by one pet cat over its lifetime: in 17 years, the desexed female brought home 558 prey, including mice, rats, rabbits, hares, weasels and birds [83]. The author did not believe that this predation had negative effects on the local wildlife.

#### USA

Few respondents in the USA considered pet cats a threat to wildlife in cities, towns and rural areas or on farms, but about half considered pet cats a threat in nature reserves. American respondents also scored the lowest of the western countries on the wildlife scale, although it may be that given the high incidence of confinement in the USA respondents had in mind that cats were not a threat because they were likely to be indoors. Respondents were ambivalent about the need for legislation regulating ownership and husbandry of pet cats, perhaps reflecting strong community divisions on the issue (see [34] for coverage of these issues as related to managing colonies of feral or semi-feral cats). On the one hand, conservation groups advocate regulations to enhance cat welfare, reduce public nuisance and protect wildlife (e.g. [38, 84]), while on the other hand lobby groups such as the Cat Fanciers' Association resist regulations they perceive as unreasonable, even offering the support of a legislative committee (http://www.cfainc.org/Legislative/LegislativeGroup.aspx). The primary motivation for much existing legislation appears to be the reduction of public nuisance (e.g. [85]). Within Hawaii, where many of the cats are free-roaming, there is strong potential for interaction with feral cat colonies as well as opportunities to depredate native wildlife, including endangered species. Thus conservation of Hawaii’s unique fauna may be important in shaping attitudes there.

Wildlife mortality from pet cats in the continental USA is estimated at 684 million birds and 1,249 million mammals annually [18], while the American Bird Conservancy [86] estimated that 500 million to one billion birds are killed each year by pet cats. As an example of effects at the level of a single species, Balogh, Ryder and others [87] determined that predation accounted for 79% of mortalities of post-fledging grey catbirds (*Dumatella carolinensis*), with 47% of these mortalities caused by domestic cats (not necessarily pets). While they acknowledged that they could not determine if this mortality was compensatory or additive, the successful development of collar-worn predation deterrents by USA businesses [88–90] shows that many cat owners in the USA wish to curtail their cats' hunting behaviour.

#### UK

Respondents from the UK were the least supportive of introducing legislation and scored lowest on the restrictions scale. They were unlikely to believe that pet cats are harmful to wildlife in towns, cities and rural areas or farms, and only 61% of non-owners and 41% of cat owners believed that pet cats are harmful to wildlife in reserves. However, the UK had the highest proportion of cats known to have hunted vertebrate prey on at least one occasion, probably because most cats are kept either inside or outside but free roaming (64%), or only confined at night (23%). Requiring owners to restrict wandering behaviour by either keeping their cats in at night or keeping them confined to their owner’s property was very unpopular amongst both cat owners and non-owners. Requiring owners to desex their cats was only supported by about 66% of owners, although the actual desexing rate was very high (93%). These results are in close accord with independent findings that UK cat owners from two small rural communities disagree that cats harm wildlife populations and are unsupportive of most cat management actions other than neutering [91]. The similarity of attitudes to those from the urban populations we surveyed suggests a characteristic position for UK citizens irrespective of place of residence. Historically, there is a strong tradition in the UK of keeping farm cats to control vermin, so responses are consistent with this view of the function of cats.

UK responses are consistent with the finding that UK citizens respond even more positively than people elsewhere to felids as symbols of nature [69]. They also match the official message from bodies such as the Royal Society for the Protection of Birds (RSPB) that '… there is no scientific evidence that predation by cats in gardens is having any impact on bird populations UK-wide. This may be surprising, but many millions of birds die naturally every year, mainly through starvation, disease, or other forms of predation. There is evidence that cats tend to take weak or sickly birds.' However, there is acknowledgment that: 'Cat predation can be a problem where housing is next to scarce habitats such as heathland, and could potentially be most damaging to species with a restricted range (such as cirl buntings) or species dependent on a fragmented habitat (such as Dartford warblers on heathland)' [92].

Studies of predation by pet cats in the UK have moved from estimates of nationwide losses based on extrapolations from local or regional mortality (e.g. [3, 93]) to assessments of population risk that support the conclusion that at least some populations are affected by cat predation [9, 36, 94], sublethal effects from cat presence [95], or cats mediating the effects of other predators [96]. However, the attitudes expressed by our UK respondents and the RSPB position endorse the opinion that 'Management of the predation behavior of urban cat populations in the UK is likely to be challenging and achieving this would require considerable engagement with cat owners' [36 pg. 1].

#### Japan

Japan was the only country where owners were more supportive of restrictions than non-owners. The cultural issues underlying this may be complex, because welfare issues such as reducing the incidence of cats being hit by cars or getting lost apply in urban environments elsewhere. Certainly, cats are very popular in Japan, with the phenomenon of 'cat cafés' where people engage directly with cats without owning them being '… a significant retail phenomenon throughout Japan, and in particular Tokyo' [97]. The prevailing views seem well-expressed in an online guide to keeping pets:

'Cat owners are required by municipal authorities "to keep the cat in such a manner so as it won't disturb other citizens."

Three basic principles of keeping cats:

- Keep your cat in a house.
- Use a collar marked with address and name of the owner.
- Have your cat sterilized.' [98]

Japan scored the lowest on the wildlife scale and respondents were unlikely to believe that cats were harmful to wildlife in any situation, although it may be that this was based on the assumption that cats were kept mainly indoors. The number of cats in Japan reported to have killed vertebrate fauna was the lowest across all countries, probably because most were confined. This may result from a high incidence of apartment living.

Studies of predation by pet cats in Japan are limited, although feral cats on offshore islands are significant predators of birds [99]. Research concentrates on stray (unowned) domestic cats in urban areas [100].

#### China

China’s biodiversity includes approximately 10% of known species (animal and plant), which is greater than Europe or North America [101]. Culturally, the Chinese have a long history of adopting a utilitarian approach to their biota, seeing them as resources first and other values second [102]. Infrastructures for sustainable use of natural resources and biodiversity conservation are still developing, but often include many staff and cover extensive geographic areas [101, 103, 104]. Long-standing cultural perspectives and changing regulatory approaches may underpin the views of Chinese respondents. Furthermore, China's size and diversity can lead to substantial regional differences in attitudes and regulations [103], emphasising that our results are restricted to the particular urban populations we surveyed.

While approximately 70% of owners and 80% of non-owners in China agreed that there was a need for cat legislation, their scores on the restriction scale were similar to New Zealand, the USA and Japan. Perhaps the Chinese respondents did feel that there should be cat legislation, but not in the areas addressed in the survey. Animal welfare organisations are recent in China, with Animals Asia founded in 1998 and the Chinese Animal Protection Network (CAPN) commencing in 2004. They oppose eating cat and dog meat and support trap-neuter-return (TNR) programs to control cat numbers [105, 106]. Possibly, these are priority areas for legislation in the minds of Chinese citizens. While most Chinese respondents felt that wildlife is important in towns, cities and rural areas, they did not score highly on the wildlife scale. Chinese respondents were more likely than those from the UK, the USA and Japan to believe that pet cats endanger wildlife in cities, towns and rural areas, but less likely than people in these countries to believe they might affect wildlife in nature reserves.

### International Differences in Cat Husbandry Practices

In most countries there is a link between the number of cats per household and the manner in which they are kept (e.g. solely inside, solely outside etc.). In Japan and the USA where respondents were most likely to keep their cats solely inside, households were more likely to have multiple cats. In New Zealand and the UK, where most cats had free access inside and outside all the time, households were more likely to have only one cat. It may be that in households where cats are not permitted outside and therefore do not have contact with other animals, owners have multiple cats to keep each other company when no people are home. However, in China the majority of households had only one cat regardless of how they were kept. Lepczyk, Mertig and others [32] found a positive relationship between the number of people living at a residence and the number of cats in Michigan, USA, and suggested that larger residences are more likely to have children who own pets. This trend may occur elsewhere, but it would not account for the very high numbers of cats in many households in Japan.

Whether cats were allowed outside or not may also be related to urban density and perhaps to the likelihood of cats encountering dogs, traffic or other urban disturbances, or predators such as red foxes *Vulpes vulpes* or coyotes *Canis latrans* that enter cities or urban fringes. In Australia, significantly more cats were kept inside in Sydney, the larger city, than Wollongong. Similarly, in the USA cats in the large, mainland cities of Chicago and Los Angeles were more likely to be confined than those in Hawaii. Climate is not a factor, because Wollongong and Sydney have similar climates while Chicago and Los Angeles are very different (Table 1). Ironically for wildlife protection, while the less dense cities provide more urban gardens offering shelter and food for wildlife, the lower incidence of cat confinement may provide more opportunities for pet cats to encounter wildlife.

Of all the English-speaking countries in the survey, respondents in the USA were the most likely to keep their cats solely indoors (mainland USA 66%, Hawaii 56%). High rates of confinement between 30% and 60% are also reported in other North American studies (e.g. [39, 45, 107]). Given that the American Veterinary Medical Association, the Humane Society of the USA [108], the American Association of Feline Practitioners [109], the American Bird Conservancy [38, 45, 84, 86] and the Wildlife Society [110] support home confinement of pet cats in urban and suburban areas, professional endorsement of the practice may be important in its acceptance. Rochlitz [108] and the American Association of Feline Practitioners [109] also support enriching the indoor environment for cats. We found the highest incidence of confinement in Japan, possibly as a result of high urban densities, apartment living, regulation, and advice on responsible pet ownership [98].

Predictably, there is an association between how pet cats live (solely inside, solely outside, etc.) and whether they have ever been known to catch vertebrate prey. New Zealand and the UK, where cats were most likely to be free-roaming, recorded the most cats that have brought prey home at least once. Records of cats hunting were lowest in the mainland USA and Japan, where cats are predominantly kept inside. In Australia, Hawaii and China, partial confinement is more popular, so many cats have access outside at least some of the time. Although these cats may not hunt regularly, they still returned some prey.

There were high desexing rates of cats in all countries except China. Chinese respondents scored very low on the desexing scale and support for desexing cats that are not owned by breeders was also low. Only 43% of cats in Beijing and no cats in Harbin were desexed. These figures may reflect people considering they ‘own’ colony cats, or a cultural aversion to desexing. Considering that 45% of Chinese cats in our sample were allowed to wander away from their owner’s property at least some of the time, there are likely to be many unwanted kittens.

Despite widespread desexing of cats in countries other than China, the proportion of people who agreed with the item ‘except for a cat owned by a breeder, all cats should be desexed’ was much lower than the actual desexing rate amongst respondents' cats. For example, Japanese respondents were unlikely to agree that all cats should be desexed, but desexing rates were still high (91%). Even though cat owners choose to desex their pets, they are less likely to agree that everyone should be required to do so, despite being more supportive of compulsory desexing than non-owners (except in China).

Overall, the pattern of practices varies considerably across countries in response to a complex of environmental conditions and cultural attitudes, which we have described but not explained.

### Representativeness of the Survey

Despite the low response rates, there was little detectable evidence of survey bias. New Zealand, Japan and the UK were the only countries where cat-owners were over-represented in the survey compared to estimates in the general population, although this does assume that the published figures for cat ownership are accurate. In the case of Japan, the disparity may be an artefact of distributing questionnaires through veterinary clinics and local shops. This may also be a reason why the mean ages of Japanese respondents were much younger than those reported in other countries and could mean that the survey missed an older demographic. The possibility that cat owners were more strongly motivated to contribute could also be a factor in Japan and elsewhere.

Further support for the representativeness of the survey comes from the broad similarity of our findings with others conducted in similar communities. For example, our findings about the reluctance of UK cat owners to take any action other than desexing their pets agrees closely with studies by McDonald, Maclean and others [91] and Thomas, Fellowes and others [36]. In Australia, which has had multiple surveys of attitudes toward cats this century, our finding that 62% of owners accepted that cats killing wildlife were a problem in cities, towns and rural areas was similar to findings of 50% in Grayson, Calver and others [30] and 63% in Lilith, Calver and others [31], both for a similar demographic. In New Zealand, our results are similar to those from New Zealand market research company UMR Research's 2013 survey on public attitudes toward cats in New Zealand [111]. For example, after being prompted with figures on the number of native birds killed by cats in New Zealand, 54% of UMR respondents supported some form of control that would reduce the future population of cats (*cf*. 55% of all respondents in our survey agreeing, without prompting, that some form of cat legislation was necessary). In the UMR survey 62% of respondents believed that all pet cats should be desexed, while in our study nearly 80% of all New Zealand respondents supported the less restrictive position that, with the exception of licensed breeders, all pet cats should be desexed.

There were significant differences in the demographics of people who responded by mail or online in New Zealand and the USA, as well as differences in their responses to some questions. Mail respondents were older and more likely to be retired in both countries. Thus it was worthwhile to offer a mail survey alternative as opposed to providing only an online option, because otherwise we would have missed a significant portion of the older demographic. The variations in responses to some questions in mailed responses relative to internet responses reinforce the importance of offering the option of a mailed response.

Overall, although we have no detectable evidence of non-response bias, we believe the most likely biases in our data are: (i) over-representation of affluent people in the Western countries (an acknowledged issue with internet surveys, although such affluent people may be more likely to enter social debate or have political influence [69]; (ii) despite the offer of a mailed response to those invited to respond online, possible under-representation of older people; (iii) over-representation of responsible cat owners, as suggested by the high rates of desexing in their animals. Moreover, our results cannot be claimed to be representative of rural populations, or of socio-economic groups within cities other than our target demographic.

## Implications for Wildlife Conservation

Empirical research from Australia [17], New Zealand [7], the USA [32] and the UK [36] has established that predation by pet cats threatens at least some elements of urban or rural wildlife. While uncertainty remains regarding the risk to populations of particular species in specific localities, a precautionary approach to cat ownership and husbandry is justified while research is undertaken [112, 113]. Our chosen middle class demographic represents people most likely to be politically engaged and therefore potentially willing to engage in debate over cat husbandry [69]. Therefore their views are significant.

Of the nationalities we surveyed, Australians are most likely to accept a wildlife-based rationale for restrictions on cat ownership. Most owners and non-owners accept that pet cats may endanger wildlife (irrespective of whether or not the proposition is true), and are more accepting of measures to restrict cats in the interests of wildlife protection. Elsewhere, with the possible exception of New Zealand, arguing for restrictions on cats to protect wildlife may be counterproductive. This is especially true of the UK, where even non-owners are likely to discount cat predation as a threat to wildlife, legislation is unwanted, and there is very little support for confinement of pet cats.

Welfare arguments addressing responsible cat ownership represent an alternative approach to protect wildlife in countries other than Australia (and possibly New Zealand) where cat owners are unlikely to accept legislation based on wildlife protection, but may be more responsive to arguments based on cat welfare. This is the approach advocated by the American Bird Conservancy [84, 86]. Welfare-based arguments appeal to the cat-loving citizens of the UK, where even the concept of cat cafes is subject to careful welfare scrutiny [114]. While not enhancing cat welfare, predation deterrents may also appeal to owners concerned about the welfare of prey. Bells, pounce protectors, battery-powered alarms and colourful collar covers all reduce predation by cats significantly for different groups of vertebrate prey [88–90, 115–119], but do not stop all hunting. They could be promoted to reduce hunting success, especially if owners can be reassured that properly fitted safety collars are low-risk [120]. However, support for them is modest amongst owners in the UK [36], while in New Zealand the UMR Research's 2013 survey on public attitudes toward cats in New Zealand reported only 42% support for requiring all cats to wear a bell on their collar [111].

The most effective way to protect wildlife from the potential impact of pet cats and to improve cat welfare by reducing the risk of road accident trauma and fighting is to restrict cats to their owners' properties, ideally within runs so that some of the garden is safe from cat activity. Most cats in our study from Australia (Sydney), mainland USA, Hawaii and Japan were kept inside only, as were a third of cats from China. It is unclear whether this was done for reasons of cat welfare or wildlife protection, although the views of American, Japanese and Chinese owners on the impacts of cats on wildlife suggest that the motive was cat welfare. Fewer than 10% of New Zealand or UK owners confined their cats. Welfare campaigns highlighting the risks to roaming cats might increase the acceptability of confinement in the UK and New Zealand, especially if accompanied by advice on environmental enrichment requirements for indoor cats [108, 121], and the use of leash training and outdoor enclosures.

Of course, regulating pet cats will not be a panacea for wildlife protection. Although in some instances pet cats may pose a significant threat to local wildlife (e.g. [7, 36]), this is additive to many other impacts from anthropogenic mortality sources such as collisions with cars and other forms of transport, collisions with structures and windows (for birds), electrocution, pollution and over-hunting [17, 122–126]. The primary threat to wildlife near human dwellings is often habitat loss and fragmentation [77, 127, 128], while the decline in the average garden size and desire for houses with larger floor areas in many countries provide fewer resources for wildlife in urban areas [7, 129, 130, 131]. The substantial populations of unowned cats roaming in cities, sometimes fed deliberately by people, may also be a significant wildlife protection issue requiring unique approaches [4, 37, 80, 132]. Nevertheless, reducing the threat from pet cats will benefit some species and can be done while enhancing cat welfare. It is therefore an immediate and effective action that should be undertaken together with, not instead of, investigation of some of the more intractable causes of wildlife decline.

## Supporting Information

S1 Table Raw data underpinning the analysis of specific survey questions reported in Table 3. **There is a separate spreadsheet for each question.** 0 = agreement with the question, 1 = disagreement with the question.
(XLS)

S2 Table Raw data underpinning the analysis of Rasch location scores reported in Fig 3. Res loc: location scores for the restriction scale. Ster loc: location scores for the desexing scale. Wild loc: location scores for the Wildlife scale.
(XLS)
